# Blood neutrophil to lymphocyte ratio is associated with 90-day mortality and 60-day readmission in Gram negative bacteremia: a multi-center cohort study

**DOI:** 10.1186/s12879-024-09127-0

**Published:** 2024-02-23

**Authors:** Marcus Roldgaard, Thomas Benfield, Sandra Tingsgård

**Affiliations:** Copenhagen University Hospital– Amager and Hvidovre Hospital, Copenhagen, Denmark

**Keywords:** Gram-negative Bacteria, Bacteremia, Mortality, Patient readmission, Biomarker, Neutrophils, Lymphocytes

## Abstract

**Introduction:**

The Neutrophil-Lymphocyte Ratio (NLR) in blood has demonstrated its capability to predict bacteremia in emergency departments, and its association with mortality has been established in patients with sepsis in intensive care units. However, its potential concerning mortality and readmission in patients with Gram-negative bacteremia (GNB) is unexplored.

**Methods:**

This retrospective cohort study included patients with GNB between 2018 and 2022 from six hospitals in the Capital Region of Denmark. Patients who were immunosuppressed or had missing NLR values on the day of blood culture were excluded. Logistic regression models were used to analyze the association between NLR levels and 90-day all-cause mortality, while the logit link interpretation of the cumulative incidence function was used to assess the association between NLR levels and 60-day readmission. Associations were quantified as odds ratios (OR) with corresponding 95% confidence intervals (CI).

**Results:**

The study included 1763 patients with a median age was 76.8 years and 51.3% were female. The median NLR was 17.3 and 15.8% of patients had a quick sequential organ failure assessment score of two or three. Urinary tract infection (UTI) was the most frequent focus and *Escherichia coli* the most frequent pathogen. Statistically significant differences in median NLR were found by age group and pathogen, and for patients with or without hypertension, liver disease, chronic obstructive pulmonary disease, dementia, and alcohol abuse. 378 patients (21.4%) died before 90 days. 526 (29.8%) patients were readmitted to the hospital within 60 days. For each doubling of the NLR, the OR for all-cause 90-day mortality was 1.15 (95% CI, 1.04–1.27) and 1.12 (95% CI, 1.02–1.24) for 60-day readmission. Analysis of subgroups did not show statistically significant differences between groups in relation to the association between NLR and mortality. The discriminatory ability of NLR for mortality was limited and comparable to blood neutrophil or lymphocyte count, producing receiver operating characteristic curves with an area under the curve of 0.59 (95% CI, 0.56–0.63), 0.60 (95% CI, 0.56–0.65) and 0.53 (95% CI, 0.49–0.56), respectively.

**Conclusion:**

Blood neutrophil-lymphocyte ratio was associated with 90-day all-cause mortality and 60-day readmission in patients with GNB. However, the ratio has limited ability in predicting mortality or readmission.

## Introduction

Gram-negative bacteria are a common cause of blood stream infection and a substantial cause of sepsis, which remains a major global health burden and is associated with high mortality [1]. The incidence of Gram negative bacteremia (GNB) is rising globally [2]. The course of bacteremia and its evolution to sepsis is complex and unpredictable, with various factors such as inflammation, immune suppression, and circulatory abnormalities influencing its progression [3]. The unpredictability of bacteremia leaves a demand for tools for prognostication in these cases. Biomarkers are and have been subject of research to optimize detection and risk assessment in bacteremia and sepsis. Potentially useful markers have been identified, but few are used in clinical practice [4].

Neutrophil-to-lymphocyte ratio (NLR) is a calculated value obtained by dividing the number of neutrophils by the number of lymphocytes in a blood sample. Neutrophils are the first line of defense against pathogens, and their high count is associated with various inflammatory conditions [5]. Lymphopenia is observed in sepsis mainly due to apoptosis accelerated by tumor necrosis factor (TNF)-alpha and interleukin (IL)-1 [6], and is independently predictive of bacteremia [7]. As white blood cells are often measured in patients with suspected infection, it could be cost-effective to implement NLR in clinical practice.

NLR is proposed to reflect the interplay between the innate and adaptive immune systems, and it can also serve as an indicator of inflammation and stress [8]. Over the past two decades, NLR has been studied as a potential biomarker for identifying and predicting different medical conditions in various settings. Several studies have demonstrated that a high NLR is associated with higher mortality in patients admitted to intensive care units (ICU) including for sepsis and for other causes [9–17]. Additionally, studies have shown that NLR can predict bacterial bloodstream infections in emergency departments [18, 19]. However, there is no consensus on the cut-off value for NLR for either of these uses. It remains to be investigated whether NLR is associated with mortality in confirmed cases of bacterial bloodstream infections beyond the ICU.

This study aims to investigate whether high neutrophil-lymphocyte-ratios are associated with increased mortality and increased readmissions in patients with GNB.

## Methods

### Study population and data collection

The study included all adult patients with a blood culture positive for Gram-negative bacteria between 1/1 and 2018 and 28/12-2022 identified by the Department of Microbiology at University Hospital Copenhagen– Hvidovre Hospital. The Department of Microbiology at Copenhagen University Hospital– Amager and Hvidovre Hospital processes all microbiological samples from six Copenhagen University Hospitals (Amager, Bispebjerg, Bornholm, Frederiksberg, Glostrup and Hvidovre). Patients were followed for 90 days from the date of blood culture sampling or until death, which ever came first. Data was retrieved from patient files and kept in a Redcap database [20, 21].

Patients who did not have NLR measured on the same day as the blood culture was drawn, and patients who suffered from immunosuppression due to neutropenia (< 1,0 × 10^9/L), untreated/uncontrolled HIV infection, use of prednisolone of more than 10 mg/day for more than 30 days, chemotherapy, immunomodulating treatment (Anatomical Therapeutic Chemical Classification System L04), immune suppression due to organ transplant, or splenectomy, were excluded. Only the first GNB episode of every patient was included in the analysis.

There was no data on paO_2_ or vasopressor use, therefore the Sequential Organ Failure Assessment was modified by giving one point for oxygen saturation between 91% and 94% and two points for saturation below 91% [22]. In circulation, one point was given for systolic blood pressure below 100 [23]. Mental status was scored like Pitt Bacteremia Score, zero for alert, one for disoriented, two for stuporous and four for comatose.

Bacteremias were classified as nosocomial if the patient had been admitted for more than 48 h before the blood culture was drawn, hospital-associated if the patient had been admitted to the hospital for more than 48 h within the 14 days prior to the blood culture being drawn, and community acquired if the blood culture was collected within 48 h of admission [24].

Primary outcome was 90-day mortality. Secondary outcome was 60-day readmission. NLR, blood neutrophil and lymphocyte count were the predictors.

### Statistical analysis

Continuous variables were reported as median with interquartile ranges, while categorical variables were reported as absolute counts and percentages.

Differences between groups were compared with the Kruskal-Wallis tests.

Directed acyclic graphs (DAGs) were constructed based on previous literature about NLR and GNB to identify possible confounders and mediators to adjust for. Age, sex, focus, acquisition of bacteremia, liver disease and cancer were identified as possible confounders. A logistic model with continuous NLR values adjusted for identified possible confounders was used. The association estimates were reported as odds ratios (OR). Estimates were reported with two-sided 95% confidence intervals (CI).

As sensitivity analysis, the association between NLR grouped by quartiles and 90-day mortality were analyzed, visualized by Kaplan-Meier plots, and tested with the log-rank test. Subgroup analyses included groups by source of infection, bacteria, acquisition, and modified SOFA and qSOFA above or below two and were analyzed separately.

To avoid the potential impact of immortal time bias on the analysis of readmission, the follow-up period started on the date of discharge. Since some patients were admitted for up to 30 days, it was necessary to shorten the follow up to 60 days to secure a complete follow up for the whole cohort. The Aalen-Johansen estimator was used to estimate the absolute risk of competing events. To evaluate the differences in absolute risk among the four quartiles of NLR, Gray’s test was used. The association of NLR and 60-day readmission was analyzed with the logit link interpretation of the cumulative incidence function with all-cause mortality as a competing risk and OR as reported estimate [25]. Directed acyclic graphs (DAGs) were drawn to identify possible confounders and mediators to adjust for. Age, sex, focus, acquisition of GNB, liver disease, cancer, and admission time prior to discharge were identified as possible confounders.

Receiver operating characteristic curves were constructed to evaluate the discriminative ability of NLR, blood neutrophil and lymphocyte counts in relation to mortality and readmission and reported as the area under the curve. 95% confidence intervals were achieved through bootstrapping.

The models’ fit was tested with Goodness-of-Fit Tests. Studentized residuals were used to identify outliers with a Bonferroni *p*-value of less than 0.05. Identified outliers were then reevaluated.

Statistical analysis was performed in R v4 [26]. All tests were two-sided and *P* values of less than 0.05 were considered significant.

## Results

**Fig. 1:**
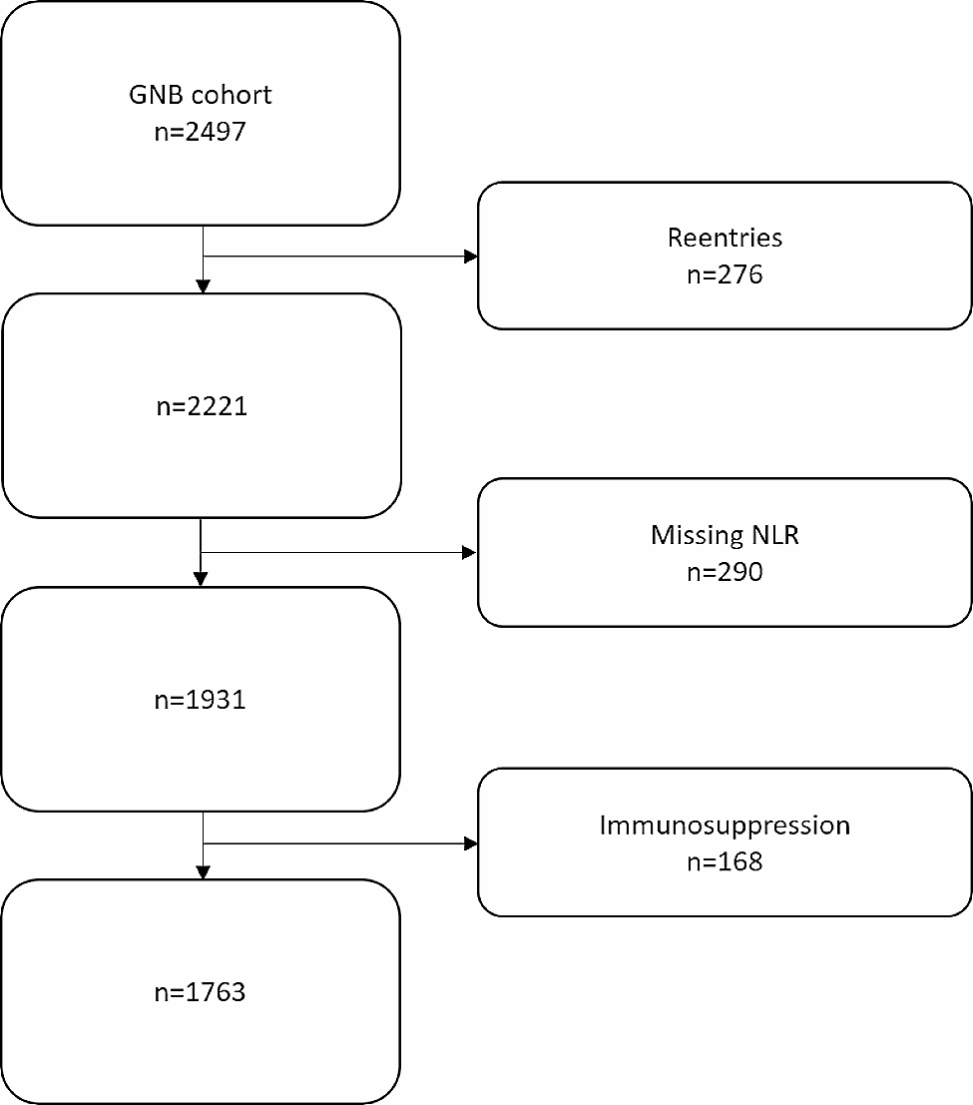
Flowchart describing the exclusion process in a cohort of patients with Gram negative bacteremia Legend: GNB: Gram negative bacteremia. NLR: neutrophil to lymphocyte ratio

There were 2497 entries in the database. After all exclusions, 1763 patients were included in the analysis (Fig. 1). The median age was 76.8 years and 51.3% were female. The urinary tract was the primary focus of infection in 70.1% of patients and the gastrointestinal system was primary focus of infection in 20.9% of patients. *Escherichia coli* (65.7%) and *Klebsiella* spp. (13.5%) were the most common pathogens in blood. Most individuals (84.2%) had a quick sequential organ failure assessment (qSOFA) score below two. About half the patients (51.0%) had a modified sequential organ failure assessment (mSOFA) score of two or above. Demographics and laboratory values for the cohort are shown in Table 1.

**Table 1:** Patient characteristics, blood neutrophil and lymphocyte count, and neutrophil to lymphocyte ratio. P refers to differences in neutrophil to lymphocyte ratio

	Total (*n* = 1763)	Neutrophils ×10^9^/L Median[IQR]	Lymphocytes ×10^9^/L Median[IQR]	NLR Median[IQR]	*P*
**Age**					< 0.001
Median [IQR]	76.8 [66.9–85.1]				
< 60 years	272 (15.4%)	11.0 [7.28–15.0]	0.90 [0.54–1.32]	13.5 [6.92–21.8]	
60–70 years	234 (13.3%)	10.6 [7.76–14.2]	0.72 [0.41–1.10]	15.4 [8.68–25.0]	
70–80 years	490 (27.8%)	11.7 [8.36–15.7]	0.69 [0.40–1.04]	17.2 [9.75–29.7]	
80–90 years	529 (30.0%)	12.0 [8.50–16.0]	0.63 [0.40–1.00]	18.9 [10.9–34.2]	
> 90 years	238 (13.5%)	13.5 [10.2–18.1]	0.60 [0.40–0.97]	23.1 [12.8–40.9]	
**Gender**					0.057
Female	904 (51.3%)	12.0 [8.73–16.0]	0.74 [0.46–1.10]	16.7 [9.36–28.8]	
Male	859 (48.7%)	16.7 [9.36–28.8]	0.64 [0.39–1.00]	18.0 [10.4–31.6]	
**Status after 90 days**					< 0.001
Alive	1385 (78.6%)	11.7 [8.11–15.0]	0.70 [0.41–1.10]	16.4 [9.29–28.6]	
Dead	378 (21.4%)	13.0 [9.30–19.0]	0.68 [0.40–1.00]	22.0 [11.8–38.3]	
**Status after 30 days**					< 0.001
Alive	1501 (85.1%)	11.8 [8.20–15.0]	0.70 [0.41–1.10]	16.8 [9.50–29.2]	
Dead	262 (14.9%)	13.6 [9.59–18.8]	0.66 [0.41–1.00]	20.9 [11.8–38.3]	
**Readmitted before 60 days**	*n* = 1578				0.002
No	1029 (65.2%)	11.3 [8.18–15.0]	0.74 [0.42–1.10]	16.2 [9.18–28.0]	
Yes	549 (34.8%)	12.4 [8.66–16.7]	0.67 [0.41–1.08]	18.6 [10.6–32.4]	
**ICU-admission during hospitalization**					0.939
No	1651 (93.6%)	12.0 [8.47–16.0]	0.70 [0.41–1.10]	17.3 [9.89–30.0]	
Yes	112 (6.4%)	11.4 [6.17–16.8]	0.62 [0.41–0.95]	17.3 [9.59–33.4]	
**qSOFA**					< 0.001
< 2	1484 (84.2%)	11.8 [8.30–15.1]	0.710 [0.43–1.10]	16.4 [9.36–28.3]	
≥ 2	279 (15.8%)	13.7 [8.85–17.0]	0.580 [0.34–0.91]	24.5 [12.3–40.0]	
**mSOFA**					< 0.001
< 2	864 (49.0%)	11.0 [8.00–14.5]	0.800 [0.50–1.20]	14.2 [8.04–25.0]	
≥ 2	899 (51.0%)	12.6 [8.60–17.0]	0.600 [0.37–0.94]	21.3 [12.3–35.7]	
**Pitt Bacteremia Score**					0.869
< 4	1691 (95.9%)	12.0 [8.38–16.0]	0.700 [0.41–1.10]	17.3 [9.76–30.0]	
≥ 4	72 (4.1%)	13.0 [8.18–16.8]	0.710 [0.40–1.10]	18.5 [10.6–33.5]	
**Source**					0.754
UTI	1236 (70.1%)	12.0 [8.71–16.0]	0.70 [0.43–1.06]	17.4 [10.0–29.7]	
GI infection	368 (20.9%)	11.0 [7.50–16.0]	0.64 [0.37–1.10]	17.8 [8.43–33.3]	
GI surgery	36 (2.0%)	10.6 [6.79–15.2]	0.59 [0.32–0.97]	17.0 [10.8–28.5]	
Pneumonia	46 (2.6%)	9.35 [6.07–14.7]	0.74 [0.40–1.12]	13.2 [8.99–26.5]	
Other	77 (4.4%)	11.8 [8.60–16.0]	0.67 [0.48–1.20]	16.5 [8.89–30.5]	
**GNB acquisition**					0.003
Community acquired	1442 (81.8%)	12.0 [8.42–16.0]	0.70 [0.41–1.08]	17.5 [10.0–30.0]	
Hospital associated	196 (11.1%)	12.0 [8.13–15.7]	0.60 [0.40–1.00]	20.1 [10.8–32.9]	
Hospital acquired	125 (7.1%)	11.2 [7.89–16.0]	0.81 [0.54–1.32]	13.8 [7.25–22.3]	
**Pathogen**					< 0.001
Escherichia coli	1159 (65.7%)	12.0 [8.40–15.3	0.70 [0.43–1.10]	16.8 [9.56–28.6]	
Enterobacter spp.	56 (3.2%)	12.3 [8.51–18.0]	0.69 [0.43–1.36]	17.5 [11.8–31.2]	
Klebsiella spp.	238 (13.5%)	12.0 [8.43–16.1]	0.70 [0.39–1.00]	18.3 [10.6–33.1]	
Proteus spp.	60 (3.4%)	12.9 [10.5–17.0]	0.55 [0.32–1.00]	22.5 [13.2–39.2	
Other	103 (5.8%)	9.10 [5.35–13.5]	0.88 [0.44–1.29]	11.8 [4.74–25.4]	
Polybacteremia with GNB	78 (4.4%)	12.7 [9.45–16.2]	0.54 [0.37–0.77]	23.7 [14.0–37.8]	
Polybacteremia with non-GNB	69 (3.9%)	12.0 [8.40–17.4]	0.57 [0.30–1.04]	22.0 [13.4–41.5]	
**Atypical bacteria**					0.124
No	1752 (99.4%)	12.0 [8.35–16.0]	0.70 [0.41–1.10]	17.3 [9.82–30.0]	
Yes	11 (0.6%)	12.4 [10.2–16.2]	0.49 [0.40–0.66]	21.6 [17.4–51.8]	
**Liver disease**					0.037
No	1693 (96.0%)	12.0 [8.50–16.0]	0.70 [0.41–1.10]	17.5 [10.0–30.3]	
Yes	70 (4.0%)	8.76 [6.12–13.8]	0.71 [0.40–1.10]	14.8 [6.86–26.2]	
**Solid cancer**					0.068
No	1519 (86.2%)	12.0 [8.32–15.6]	0.70 [0.42–1.10]	17.1 [9.69–29.8]	
Yes	244 (13.8%)	12.4 [8.58–16.9]	0.64 [0.39–1.00]	20.0 [10.6–33.9]	
**Hematologic cancer**					0.040
No	1759 (99.8%)	12.0 [8.35–16.0]	0.70 [0.41–1.10]	17.4 [9.91–30.0]	
Yes	4 (0.2%)	10.8 [7.40–13.9]	1.29 [0.72–4.21]	4.15 [2.83–10.3]	
**Hematologic disease**					0.188
No	1719 (97.5%)	12.0 [8.40–16.0]	0.70 [0.41–1.10]	17.5 [10.0–30.0]	
Yes	44 (2.5%)	10.2 [7.03–13.3]	0.68 [0.33–1.00]	13.9 [7.48–31.8]	
**Heart disease**					0.198
No	1109 (62.9%)	12.0 [8.52–16.0]	0.71 [0.42–1.10]	17.3 [9.57–29.4]	
Yes	654 (37.1%)	11.1 [8.11–16.0]	0.65 [0.40–1.00]	17.4 [10.5–32.1]	
**Cardiovascular disease**					0.500
No	1450 (82.2%)	11.9 [8.30–15.8]	0.70 [0.40–1.10]	17.4 [9.60–30.0]	
Yes	313 (17.8%)	12.1 [8.71–16.0]	0.69 [0.44–1.10]	17.1 [10.7–30.7]	
**Hypertension**					0.003
No	858 (48.7%)	11.3 [7.85–15.5]	0.72 [0.41–1.10]	16.3 [8.99–29.3]	
Yes	905 (51.3%)	12.0 [8.70–16.0]	0.67 [0.41–1.00]	18.5 [10.8–30.7]	
**Peripheral vascular disease**					0.753
No	1698 (96.3%)	12.0 [8.37–16.0]	0.70 [0.41–1.10]	17.4 [9.73–30.0]	
Yes	65 (3.7%)	11.9 [8.27–16.0]	0.67 [0.49–1.10]	17.1 [12.7–28.6]	
**Renal disease**					0.162
No	1622 (92.0%)	12.0 [8.30–16.0]	0.70 [0.41–1.10]	17.2 [9.68–30.0]	
Yes	141 (8.0%)	12.0 [9.60–16.0]	0.70 [0.41–1.10]	19.4 [11.1–32.8]	
**COPD**					0.033
No	1511 (85.7%)	12.0 [8.30–15.7]	0.69 [0.40–1.08]	17.6 [10.1–30.6]	
Yes	252 (14.3%)	12.0 [8.78–16.0]	0.76 [0.50–1.19]	15.6 [8.46–27.5]	
**Dementia**					0.046
No	1598 (90.6%)	12.0 [8.20–16.0]	0.70 [0.41–1.10]	17.1 [9.58–30.0]	
Yes	165 (9.4%)	13.0 [9.30–16.0]	0.67 [0.40–1.00]	19.7 [11.3–34.7]	
**Connective tissue disease**					0.938
No	1703 (96.6%)	12.0 [8.32–16.0]	0.70 [0.41–1.10]	17.4 [10.0–30.0]	
Yes	60 (3.4%)	12.2 [8.77–16.0]	0.76 [0.43–1.10]	15.7 [8.83–35.5]	
**Diabetes**					0.883
No	1304 (74.0%)	12.0 [8.30–16.0]	0.70 [0.41–1.09]	17.2 [10.0–30.1]	
Yes	459 (26.0%)	12.0 [8.40–16.0]	0.68 [0.41–1.10]	17.8 [9.46–30.0]	
**Alcohol abuse**					0.016
No	1620 (91.9%)	12.0 [8.40–16.0]	0.69 [0.41–1.10]	17.5 [10.0–30.5]	
Yes	143 (8.1%)	11.0 [7.58–15.0	0.80 [0.46–1.15]	15.3 [7.25–26.1]	
**Neutrophils**					
Median [IQR]	12.0 [8.35–16.0]	-	-	-	
**Lymphocytes**					
Median [IQR]	0.700 [0.410–1.10]	-	-	-	
**NLR**					
Median [IQR]	17.3 [9.85–30.0]	-	-	-	

IQR: Interquartile range, NLR: Neutrophil-lymphocyte ratio, qSOFA: Quick sequential organ failure assessment, GNB: Gram negative bacteremia, COPD: chronic obstructive pulmonary disease

The median blood neutrophil count and blood lymphocyte count were 12 and 0.7, respectively. The median NLR was 17.3. NLR increased with age, mSOFA and qSOFA score and differed by bacterial etiology and acquisition. Furthermore, it was higher in individuals who died within 30 and 90 days compared to those who survived and was higher among individuals who were readmitted within 60 days compared to those who were not readmitted. NLR was lower in patients with liver disease, hematologic cancer, chronic obstructive pulmonary disease, or alcohol abuse compared to those without. For patients with dementia or hypertension, NLR was found to be higher than in those without. NLR did not differ for those with other comorbidities when compared to patients without these comorbidities. Baseline characteristics of the excluded patients were similar to the included patients, however, the excluded patients had higher qSOFA and 90-day mortality.

### Mortality

**Fig. 2:**
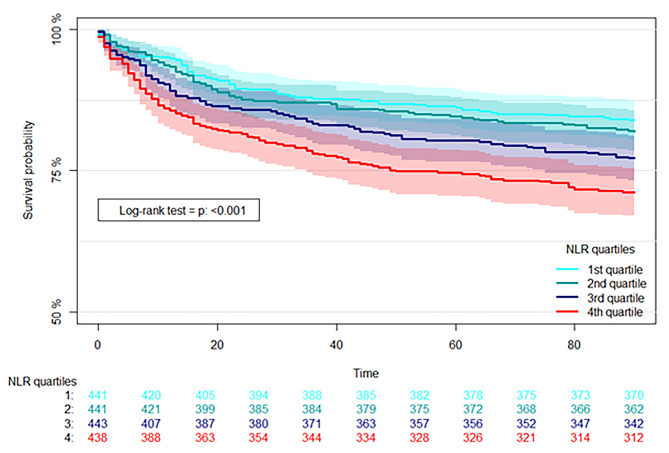
Kaplan-Meier curve depicting survival probability 90 days following positive blood culture Legend: Comparison between quartiles of neutrophil to lymphocyte ratio (NLR).

**Fig. 3:**
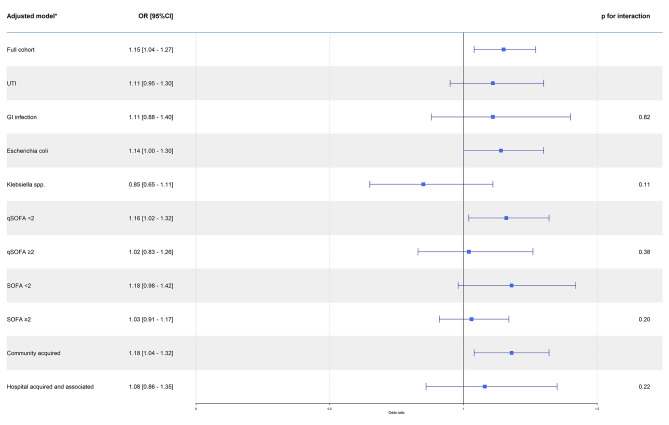
Odds ratio estimates from logistic models with log-2 transformed neutrophil to lymphocyte ratio (NLR) Legend: Adjusted by age, sex, focus, acquisition, liver disease and cancer. UTI: Urinary tract infection. GI: Gastro-intestinal. SOFA: sequential organ failure assessment. qSOFA: quick sequential organ failure assessment

Within 90 days of admission, 378 (21.4%) had died. Survival curves are shown in Fig. 2 (Log rank test: *P* < 0.001). NLR in the fourth quartile compared to the first quartile was associated with mortality (OR 1.57 (95% CI, 1.09–2.25, *p* = 0.01), whereas the second and third quartiles did not differ from the first quartile (OR 1.13, 95% CI, 0.77–1.65, *p* = 0.53 and OR 1.41, 95% CI, 0.97–2.04, *p* = 0.07). For each doubling of NLR, the adjusted OR of dying within 90 days was 1.15 (95% CI, 1.04–1.27, *p* = 0.009) (Fig. 3). Subgroups did not differ from the full cohort.

**Fig. 4:**
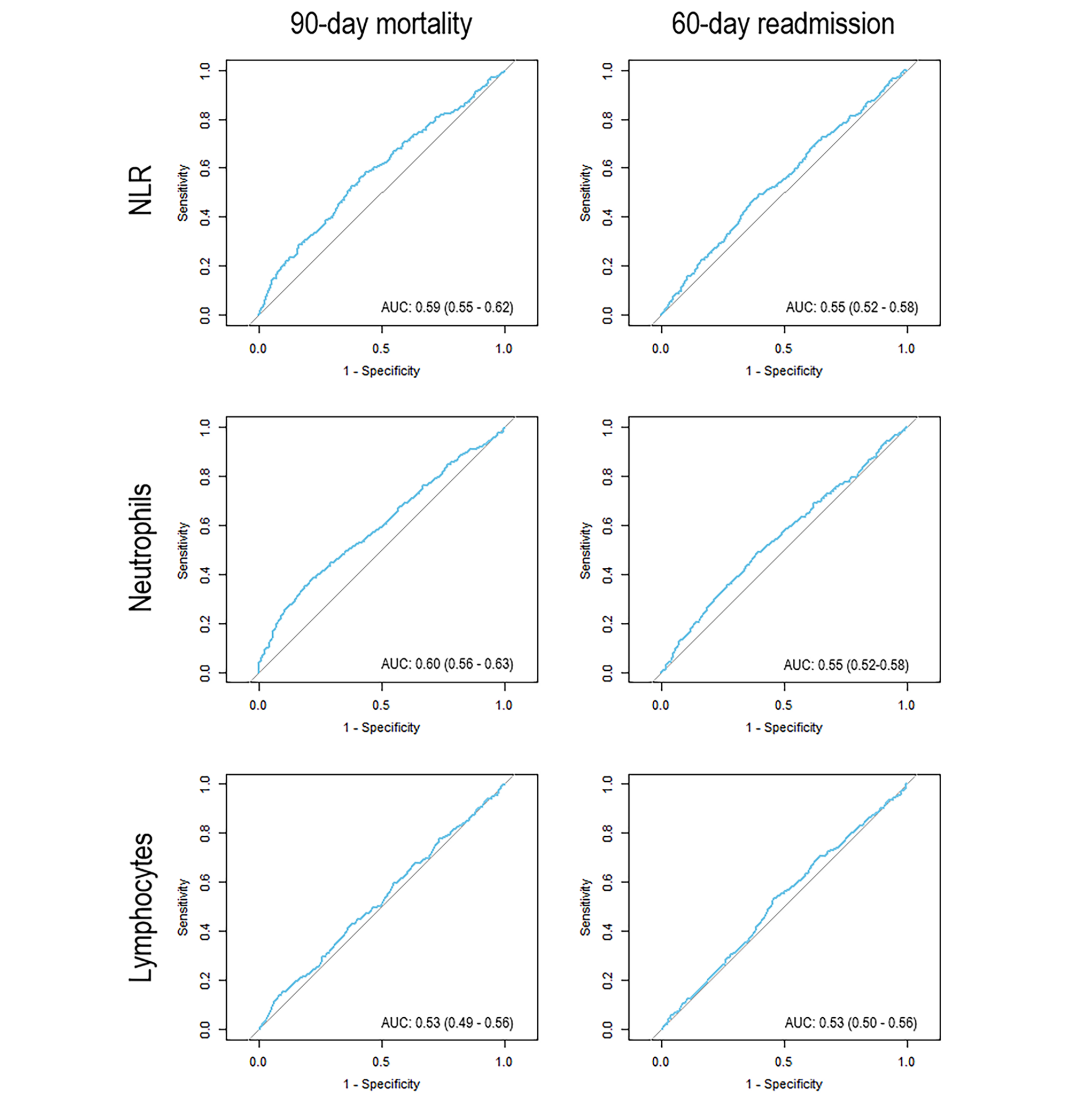
Receiver operator characteristic curves Legend: 90-day mortality and 60-day readmission predicted from neutrophil to lymphocyte ratio (NLR), blood neutrophil count and blood lymphocyte count

The discriminatory ability of NLR, blood neutrophil count and blood lymphocyte count to predict 90-day mortality was (AUCs) 0.59 (95% CI, 0.55-62), 0.60 (95% CI, 0.56–0.63) and 0.53 (95% CI, 0.49–0.56), respectively (Fig. 4). The roc-curves of NLR in subgroups did not differ from roc curves of the full cohort.

### Readmission

**Fig. 5:**
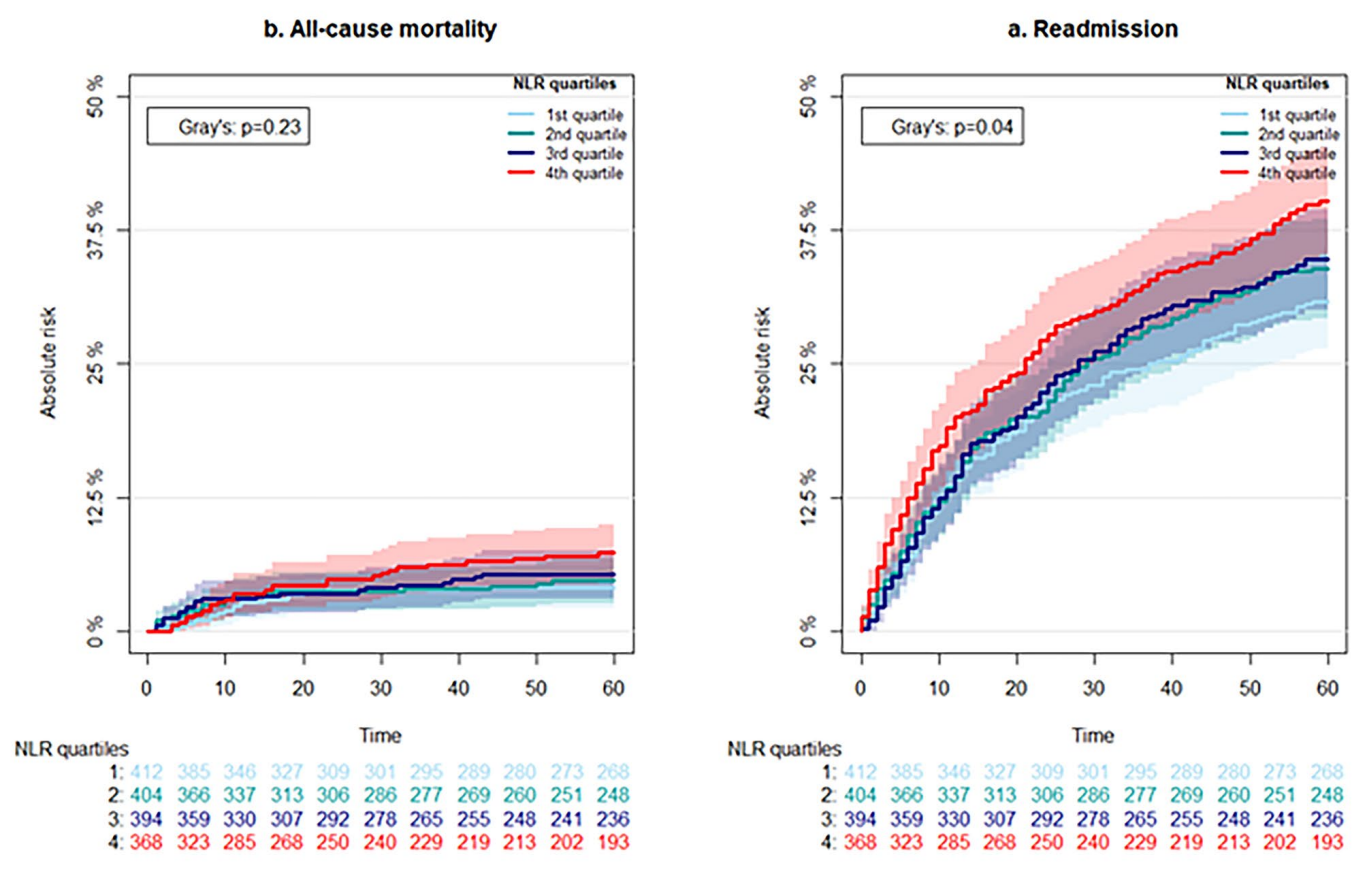
Cumulative incidence curves depicting absolute risk of being readmitted or dying after discharge from the hospital stratified by quartiles of neutrophil to lymphocyte ratio (NLR)

The readmission analysis included 1573 patients, since 185 had died before discharge and were excluded from this analysis. Within the 60 days of follow up, 526 (29.8%) patients were readmitted to the hospital. The cumulative incidence curves by NLR quartile are shown in Fig. 5. Admission rates were different between NLR quartile groups, with a *p*-value of 0.04. The 60-day readmission odds ratio was 1.12 (95% CI, 1.02–1.24, *p* = 0.02) per doubling of NLR.

The AUC of the ROC-curves predicting 60-day readmission based on NLR, blood neutrophil count and blood lymphocyte count was 0.55 (95% CI, 0.52-58), 0.54 (95% CI, 0.52–0.57) and 0.53 (95% CI, 0.50–0.56), respectively (Fig. 4).

## Discussion

In the present study, we found an association between NLR and 90-day all-cause mortality as well as an association between NLR and readmission within 60 days in a cohort of patients with GNB. However, NLR had poor discriminatory ability for mortality and readmission.

The association found between mortality and NLR confirms the findings of most previous studies. Three studies have compared NLR quartiles or quintiles and their association to mortality. Salciccioli et al. found an association between NLR and 28 day mortality in a cohort of ICU patients but the association did not persist in their sepsis subgroup [9]. Hwang et al. compared NLR quintiles in a Cox model and found higher hazard ratios of 28 day mortality in the first and fifth quintiles compared to the third quintile [11], while Ye et al. found stepwise increasing odds ratios across quartiles similar to our results [12]. Hwang et al. did not have an exclusion criterion for immunosuppression and their first quintile had a median NLR of 0.2, which may suggest this group had neutropenia - a condition that would have caused exclusion in our study. Other studies [13, 14, 27] have constructed models comparing two groups based on a predetermined cutoff. These studies also show an association between high NLR and mortality, although direct comparison is difficult due to different statistical approaches, along with the fact that the cohort in this study may need a different cutoff because of the high NLR values measured.

Subgroup models did not show any significant differences between groups in relation to the association between NLR and mortality, although it is interesting, that the association seems larger in patients with lower modified SOFA or qSOFA compared to patients with higher modified SOFA or qSOFA. This could be investigated in larger studies.

Conducting an analysis of 90-day mortality, as opposed to solely focusing on a short-term mortality, holds significance in the investigation of the association between NLR and outcomes in cases of bacteremia and sepsis, as the extended timeframe provides a more comprehensive understanding of a potential longer lasting impact on patient outcomes. This extended timeframe also allowed us to find an increased OR of being readmitted with higher NLR levels at the time of blood culture draw.

To our knowledge, this study is the first to investigate the association between readmission after discharge and NLR levels. The association aligns with theories proposed by Venet and Monneret [28] and Finfer et al [29], that bacteremia and sepsis can cause lymphopenia and prolonged immunosuppression in the aftermath. Unfortunately, we only have data on NLR levels at the time of the initial blood culture, but it would have been interesting to see if an NLR value measured on the day of discharge would be even better at identifying patients at risk of being readmitted.

In this study, NLR was not found to be a good predictor of mortality or readmission. The performance for NLR and mortality is comparable to the performance in some studies [12–15], while other studies found better performance than in our study [10, 16, 17]. These latter studies had smaller cohorts, which may have had more selection, compared to our study population, which could explain the discrepancy.

This study found a surprisingly high median NLR of 17.3, contrasting median NLR values ranging between 6.7 and 11.1 observed in previous studies investigating NLR and mortality in sepsis or bacteremia patients [9, 11–13, 27, 30]. Considering the results of this study and previously mentioned NLR studies, high disease severity and mortality rates should be associated with high NLR values. The NLR values in this cohort are therefore unexpectedly high when fewer had sepsis (51.0% with modified SOFA ≥ 2) and the 30-day mortality was lower (14.9%) compared to cohorts consisting of purely septic patients or patients in critical care with 30-day mortality rates of 20–35%. The disparity in these findings could potentially be attributed to higher age. While the median age in the present study was 76.8, previous studies included patients with median ages in the range of 60 to 74. This explanation finds support in the results of the study at hand, which indicated a statistically significant association between age and NLR values. Another plausible explanation is that this cohort consists exclusively of patients with GNB. Gram negative bacteria are covered with lipopolysaccharide (LPS), also known as endotoxin, which has been shown to cause a rise in neutrophil count and a fall in lymphocyte count upon experimental infusion [31]. Thirdly, as leukocyte counts in infected patients are highly dynamic, the timing of the blood sampling is an important factor in which values are measured. This fact is also important to consider in the comparison of results between studies, as some studies measure at admission to ICU, while the present study use a sample on the day of blood culture.

This study possesses notable strengths, primarily the fact that this is a multicenter study with complete follow up. The inclusion of consecutive patients with a blood culture positive for Gram negative bacteria gives data on a broad range of patients with different etiology and severity. These strengths make our results robust and generalizable.

There are some limitations to consider in the interpretation of the results in this study arising from its retrospective observational nature. Since the patients were treated by doctors unaware of our intention to investigate NLR, there may be a selection bias in which of the patient had differential counts performed on their blood samples, and which patients had not. If anything, this bias may have caused an underestimation since the excluded patients had higher 90-day mortality and qSOFA scores than those included. There is a chance of unmeasured confounding, which we can’t account for. The fact that we only have data from one differential count is also a limitation due to the highly dynamic nature of different populations of leukocyte counts.

In conclusion, increased NLR on the day of blood culture sampling was associated with 90-day mortality and 60-day readmission in patients with GNB. It did not prove to be useful for predicting either of the outcomes.

## Data Availability

All data available by reasonable request to corresponding author.

## Abbreviations

AUC: Area under the curve
CI: Confidence interval
DAG: Directed acyclic graphs
GNB: Gram negative bacteremia
ICU: Intensive care unit
IL: Interleukin
IQR: Interquartile range
LPS: Lipopolysaccharide
mSOFA: Modified sequential organ failure assessment
NLR: Neutrophil-Lymphocyte ratio
OR: Odds ratio
qSOFA: Quick sequential organ failure assessment
SOFA: Sequential organ failure assessment
TNF: Tumor necrosis factor
UTI: Urinary tract infection
